# Aberrant Splicing Promotes Proteasomal Degradation of L-type Ca_V_1.2 Calcium Channels by Competitive Binding for Ca_V_β Subunits in Cardiac Hypertrophy

**DOI:** 10.1038/srep35247

**Published:** 2016-10-12

**Authors:** Zhenyu Hu, Jiong-Wei Wang, Dejie Yu, Jia Lin Soon, Dominique P. V. de Kleijn, Roger Foo, Ping Liao, Henry M. Colecraft, Tuck Wah Soong

**Affiliations:** 1 Department of Physiology, Yong Loo Lin School of Medicine, National University of Singapore 117597, Singapore; 2 Department of Surgery, Yong Loo Lin School of Medicine, National University of Singapore, 117597, Singapore; 3 Cardiovascular Research Institute, National University Health Systems, Centre for Translational Medicine, 117599, Singapore; 4 National Heart Centre Singapore, 5 hospital drive, 169609, Singapore; 5 Dept of Cardiology, University Medical Center Utrecht, 3584CX Utrecht, The Netherlands; 6 Calcium Signaling Laboratory, National Neuroscience Institute, 11 Jalan Tan Tock Seng 308433, Singapore; 7 Department of Physiology and Cellular Biophysics, Columbia University, College of Physicians and Surgeons, New York, NY 10032, USA; 8 NUS Graduate School for Integrative Sciences and Engineering, 117456, Singapore; 9 Neurobiology/Ageing Programme, National University of Singapore, 117456, Singapore

## Abstract

Decreased expression and activity of Ca_V_1.2 calcium channels has been reported in pressure overload-induced cardiac hypertrophy and heart failure. However, the underlying mechanisms remain unknown. Here we identified in rodents a splice variant of Ca_V_1.2 channel, named Ca_V_1.2_e21+22_, that contained the pair of mutually exclusive exons 21 and 22. This variant was highly expressed in neonatal hearts. The abundance of this variant was gradually increased by 12.5-folds within 14 days of transverse aortic banding that induced cardiac hypertrophy in adult mouse hearts and was also elevated in left ventricles from patients with dilated cardiomyopathy. Although this variant did not conduct Ca^2+^ ions, it reduced the cell-surface expression of wild-type Ca_V_1.2 channels and consequently decreased the whole-cell Ca^2+^ influx via the Ca_V_1.2 channels. In addition, the Ca_V_1.2_e21+22_ variant interacted with Ca_V_β subunits significantly more than wild-type Ca_V_1.2 channels, and competition of Ca_V_β subunits by Ca_V_1.2_e21+22_ consequently enhanced ubiquitination and subsequent proteasomal degradation of the wild-type Ca_V_1.2 channels. Our findings show that the resurgence of a specific neonatal splice variant of Ca_V_1.2 channels in adult heart under stress may contribute to heart failure.

Cardiac excitation-contraction coupling is mainly initiated by Ca^2+^ influx through L-type voltage gated Ca_V_1.2 channels in cardiomyocytes via Ca^2+^-induced Ca^2+^ release mechanisms^1^. The Ca_V_1.2 channel comprises a pore-forming α_1_ subunit and auxiliary α_2_δ and β subunits^2^. The accessory subunits modulate the channel biophysical properties and are involved in the anchorage, trafficking and post-translational modification of the pore-forming α_1_ subunit^3^. In particular, the Ca_V_β subunit was recently reported to promote the trafficking of Ca_V_1.2 channels to the plasma membrane by inhibiting the proteasomal degradation of the channels^4^. Genetic deletion of either the pore-forming α_1_ subunit or Ca_V_β subunit led to embryonic death with cardiac defects^5^,^6^.

In cardiac hypertrophy and heart failure, linkage to alteration in Ca^2+^ influx via Cav1.2 channels has been controversial^7^,^8^. Clinical trials using Ca^2+^ channel blockers for heart failure have been disappointing with either no beneficial effects or a worse outcome of reduced ejection fraction^9^,^10^,^11^. Nevertheless, in human failing cardiomyocytes the density of Ca_V_1.2 channels was decreased compared to normal cardiomyocytes^12^. In line with these findings, decreased Ca_V_1.2 channel activity was recently reported to induce cardiac hypertrophy and heart failure in genetically modified mice^8^. More importantly, the hypertrophied cardiomyocytes induced by pressure overload showed drastic decrease in Ca_V_1.2 channel density and activity due to reduced expression of the Ca_V_1.2 channels. The mechanisms, however, by which the density and activity of Ca_V_1.2 channels were reduced is unknown.

The pore-forming α_1_ subunit undergoes extensive alternative splicing that potentially generates multiple functionally diversified Ca_V_1.2 variants in human^13^ and rodent hearts^14^. Alternative splicing could be developmentally regulated^14^,^15^ and involved in myocardial infarction^16^ and heart failure^17^. In human diseases, alternative splicing of α_1_ subunit has been reported in failing human ventricular cardiomyocytes and atherosclerotic human arteries^17^,^18^. Ectopic expression of some alternative splicing variants modulated the expression and activity of the Ca_V_1.2 channels^5^,^14^. In the present study, we identified a Ca_V_1.2 splice variant containing the mutually exclusive exons 21 and 22 (e21 + 22), named Ca_V_1.2_e21+22_ channel, which was highly expressed in neonatal and hypertrophied adult hearts. As the newly identified channel variant does not conduct Ca^2+^ ions, we hypothesized that it may account for the reduced expression and activity of Ca_V_1.2 channels in hypertrophied cardiomyocytes induced by pressure overload^14^.

## Results

### Differential expression of alternatively spliced isoforms of Ca_V_1.2 channels in neonatal versus adult rat hearts

**Figure 1:**
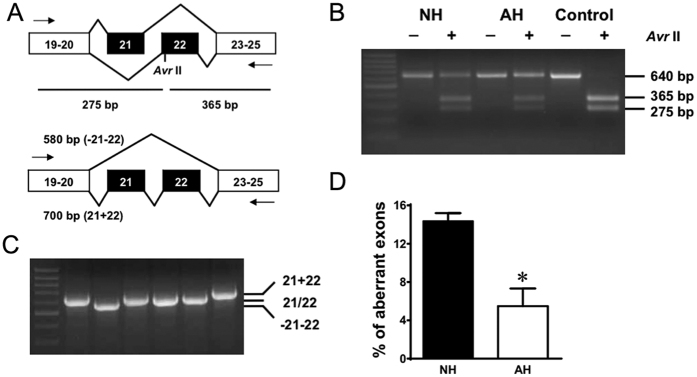
Inclusion level of exons 21+22 in Ca_V_1.2 channels in neonatal hearts is higher than that in adult hearts. (**A**) Exons 21 and 22 are mutually exclusive exons. RT-PCR across exons 19–25 could generate a fragment of 640 bp. Digestion with AvrII could produce two smaller fragments of 275 bp and 365 bp. Besides, aberrant exclusion or inclusion of both exons 21 and 22 would generate two fragments of 580 bp and 700 bp, respectively. (**B**) mRNA of Ca_V_1.2 channels from both neonatal (NH) and adult (AH) hearts showed partial digestion by AvrII. Complete digestion was observed in control DNA containing exon 22. (**C**) Colony screening of a neonatal heart identified bands of three size classes: 580 bp, 640 bp and 700 bp. (**D**) Summary of aberrant splicing rate in rat neonatal hearts (NH, n = 5) and adult hearts (AH, n = 5). Data were shown as mean ± SEM. *p < 0.05.

Mutually exclusive exons 21 and 22 encode the IIIS2 transmembrane segment and part of the linker region between IIIS1 and IIIS2. Restriction enzyme AvrII digests within exon 22 only, but not exon 21 (Fig. 1A). RT-PCR across exons 19 to 25 produced a fragment of 640 bp in length. Control cDNA containing exon 22 only was completely digested by Avr II. Under similar conditions, however, only a portion of the RT-PCR products from both neonatal and adult hearts were digested, suggesting the presence of a mixture of PCR products expressing exon 21 and exon 22 in four possible combinations of e21, e22, e(21 + 22) and ∆e(21 + 22) (Fig. 1B). The predicted PCR product sizes are 640 bp for e21 or e22, 700 bp for e(21 + 22) and 580 bp for ∆e(21 + 22) (Fig. 1C). The results were confirmed by sequencing the PCR products. Inclusion of both exons will generate a channel with one additional transmembrane segment and may result in a drastic change in the topology of the channel. In this study, we focused on the splice variant including both exons e(21 + 22): Ca_V_1.2_e21+22_ channels. Transcript-scanning demonstrated that the abundance of Ca_V_1.2_e21+22_ channels in rat neonatal heart (14.3%) was 2.5 times higher than that in adult heart (5.5%, *P* = 0.0124, Fig. 1D).

### Increased abundance of Ca_V_1.2_e21+22_ channels in hypertrophied heart

**Figure 2:**
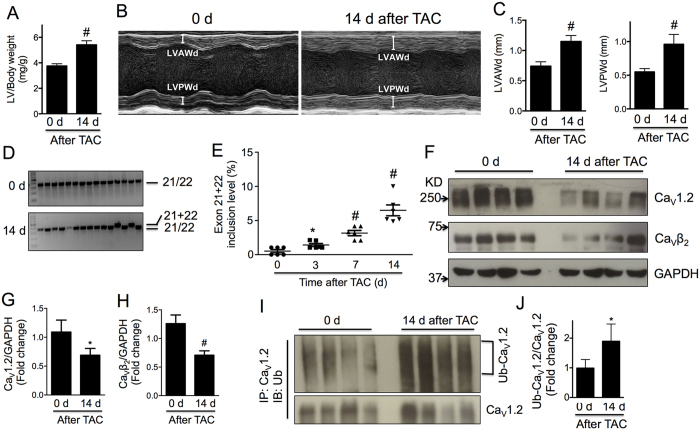
Abundance of cardiac Ca_V_1.2_e21+22_ channels is increased in mice in response to TAC surgery. (**A**) Increased ratio of left ventricle to body weight in TAC mice. (**B**) Representative M-mode echocardiography images of mouse hearts before and 14 days after TAC surgery indicating progression of cardiac hypertrophy. (**C**) Increased LVAWd and LVPWd in TAC mice. (**D**) Representative gel photos for transcript screening of exons 21 + 22 inclusion level. Each lane represents a single colony expressing exons 21 + 22 or exon 21/22. (**E**) Inclusion level of exons 21 + 22 increased from 0.52% to 6.49% with the development of cardiac hypertrophy induced by pressure overload within 14 days (n = 6). (**F**–**H**) Expression levels of total Ca_V_1.2 channels and Ca_V_β_2_ subunits in left ventricles (n = 8). (**I**,**J**) Ubiquitination level of cardiac Ca_V_1.2 channels in left ventricles (n = 8). Data were shown as mean ± SEM. *p < 0.05, ^#^*p* < 0.01. 1-way ANOVA was performed for multiple comparisons in panel E.

Alteration in the expression of developmentally regulated Ca_V_1.2 splice variants has been implicated in cardiac hypertrophy^19^ and heart failure^17^. To examine whether the alternative splicing isoform Ca_V_1.2_e21+22_ in neonatal heart reemerges in the hypertrophic adult heart, we performed transverse aortic constriction (TAC) surgery on the mice as done previously^8^ to generate pressure-overload induced cardiac hypertrophy that gradually develops and reaches a peak on day 14 after TAC surgery^20^. As expected, left ventricular (LV) weight to body weight, measured in isolated ventricles, increased significantly after two weeks of TAC (Fig. 2A). Thickening of left ventricular anterior and posterior walls at end diastole (d) or end systole (s) was overt via echocardiography (Fig. 2B,C and Table 1). Cardiac hypertrophy was also evidenced by the gradual increase in Myh7 and decrease in Myh6 at mRNA level (Supplementary Fig. S1). Furthermore, the heart rate of those mice was significantly increased while cardiac function was depressed as indicated by the reduction in ejection fraction and fraction shortening (Table 1).

In the hypertrophied heart, we first examined the expression level of Ca_V_1.2 channels. Consistent with Goonasekera’s report^8^, total protein level of α_1_ subunit of Ca_V_1.2 channels was reduced by 40% in mouse left ventricles 14 days after TAC surgery (*p* < 0.05, Fig. 2F,G). In addition, protein level of Ca_V_β_2_ subunit was reduced by 45% (*p* = 0.01, Fig. 2F,H). Compared to baseline, the ubiquitination of Cav1.2 channels was clearly enhanced in the hypertrophic ventricles (*p* < *0*.*05*, Fig. 2I,J) indicating the involvement of proteasomal degradation. Alternative splicing of Cav1.2 channels or reemergence of fetal splicing isoforms has been suggested in stressed and failing hearts^17^,^19^, we therefore hypothesized that the neonatal isoform Ca_V_1.2_e21+22_ may reemerge in the adult heart in pressure overload induced hypertrophy and subsequently disturb the expression level of normal Ca_V_1.2 channels. To test this hypothesis, transcript-screening was performed in isolated left ventricles subjected to TAC. As shown in Fig. 2D,E, the abundance of the neonatal isoform Ca_V_1.2_e21+22_ gradually increased by approximately 12.5-folds in the left ventricles, from 0.52% to 6.49% of total Ca_V_1.2 channels, in 14 days of chronic pressure overload. In human hearts, the abundance of exons 21 + 22 was also significantly higher in the left ventricles from patients with dilated cardiomyopathy (DCM) than that from healthy donors (by 2.8 folds), but no elevation of exons 21 + 22 inclusion was observed in heart tissue from patients with ischemic cardiomyopathy (ICM, supplementary Fig. S2 and Table S1).

### Functional characterization of Ca_V_1.2_e21+22_ channels

**Figure 3:**
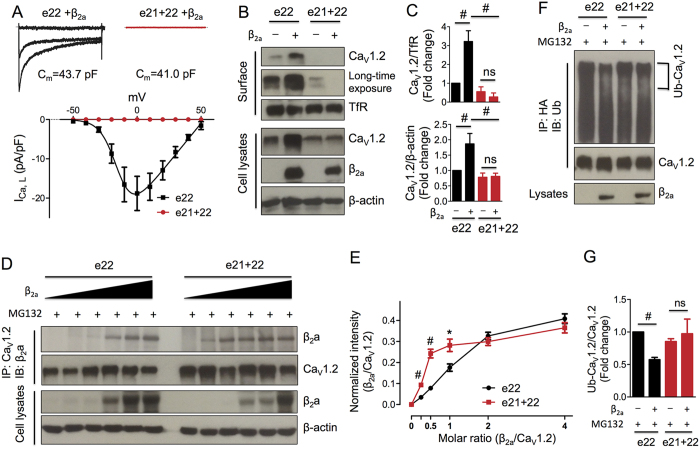
Characterization of exons 21 + 22-containing Ca_V_1.2_e21+22_ channels. (**A**) Ca_V_1.2_e21+22_ channels were co-expressed with β_2a_ and α_2_δ subunits in HEK293 cells. Whole cell patch-clamp recordings were performed on the cells expressing wild-type (n = 7) or **Ca**_**V**_**1**.**2**_**e21+22**_ (n = 8) channels. (**B**,**C**) Detection and quantification of surface and total HA-Ca_V_1.2_e22_ or Ca_V_1.2_e21+22_ channels in the presence or absence of β_2a_ subunit in transfected HEK293 cells (n = 4). Surface channels were biotinylated as indicated in the Methods. (**D**,**E**) Detection and quantification of β_2a_ subunits bound to HA-Ca_V_1.2_e22_ or Ca_V_1.2_e21+22_ channels. β_2a_ subunits were co-transfected with channels at different molar ratios of 0, 1/4, 1/2, 1/1, 2/1 or 4/1 (β_2a_/Ca_V_1.2) in HEK293 cells treated with MG132 (2.5 μM, n = 4). (**F**,**G**) Detection and quantification of the ubiquitination levels of HA-Ca_V_1.2_e22_ and Ca_V_1.2_-e21+22_ channels in transfected HEK293 cells treated with MG132 (n = 3). e22, HA-Ca_V_1.2_e22_ channels. e21 + 22, Ca_V_1.2_e21+22_ channels. Data were shown as mean ± SEM, ns, non-significant, *p < 0.05, ^#^*p* < 0.01.

To understand the pathological significance of Ca_V_1.2_e21+22_ in hypertrophied heart, we characterized this isoform *in vitro* by heterologous expression in HEK 293 cells that do not have endogenous Ca_V_1.2 channels. Compared to the robust *I*_*Ca*_ recorded from wild-type HA-tagged rat Ca_V_1.2_e22_ channels (−18.8 ± 3.6 pA/pF at 0 mV), no currents were detected from Ca_V_1.2_e21+22_ channels (Fig. 3A). Cellular localization of Ca_V_1.2_e21+22_ channels was examined by expression of α_1_ subunit with or without β_2a_ subunit in HEK 293 cells followed by surface protein biotinylation. Consistent with a previous report^4^, co-expression of β_2a_ subunit increased the surface expression level of wild-type HA-Ca_V_1.2_e22_ channels by 3.2-fold and the total expression level by 1.8-fold (Fig. 3B,C). However, Ca_V_1.2_e21+22_ channels were nearly undetectable at the cell surface regardless of the expression of β_2a_ subunit. Instead, the channel proteins were retained intracellularly (Fig. 3B,C). Of note, the total protein level of Ca_V_1.2_e21+22_ channels was much lower than that of wild-type channels, indicating that intracellular degradation may have occurred. Together, these data suggest that β_2a_ subunit failed to facilitate trafficking of Ca_V_1.2_e21+22_ channels to the cell surface.

To examine whether the intracellular retention of Ca_V_1.2_e21+22_ channels was caused by attenuated binding to β_2a_ subunit, Ca_V_1.2_e21+22_ channels were co-transfected with β_2a_ subunit at different molar ratios of 0, 1/4, 1/2, 1/1, 2/1 or 4/1 (β_2a_/Ca_V_1.2) in HEK 293 cells in the presence of a proteasomal inhibitor, MG132 (2.5 μM). In contrast to our initial hypothesis, more β_2a_ subunits were co-immunoprecipitated by Ca_V_1.2_e21+22_ than the wild-type HA-Ca_V_1.2_e22_ channels at the molar ratios of 1/4 (*p* = 0.0002), 1/2 (*p* = 0.009), and 1/1 (*p* = 0.048) (Fig. 3D,E). Furthermore, the ubiquitination level of Ca_V_1.2_e21+22_ channels did not show significant difference in the presence or absence of β_2a_ subunit (*p* = 0.65). This is in contrast to ubiquitination of wild-type channels which was drastically reduced upon co-expression with β_2a_ subunit (*p* = 0.007, Fig. 3F,G). Taken together, the non-functional Ca_V_1.2_e21+22_ channels showed stronger interactions with β_2a_ subunit and its vulnerability to ubiquitination is not ameliorated by β_2a_ subunit.

### Ca_V_β subunits did not enhance total expression of Ca_V_1.2_e21+22_ channels

**Figure 4:**
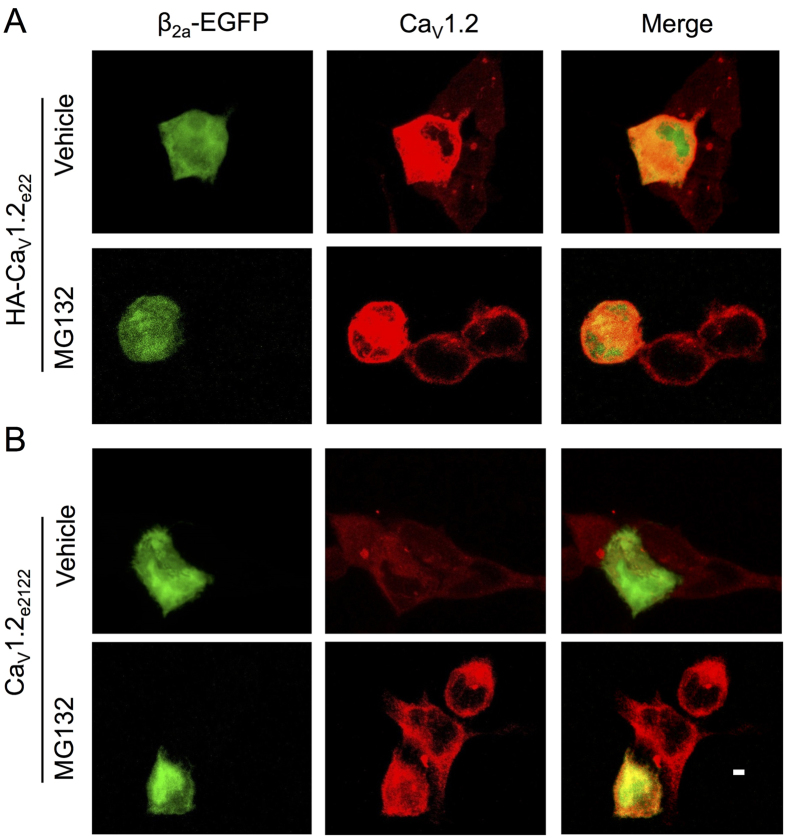
Ca_V_β subunits do not enhance the total expression of Ca_V_1.2_e21+22_ channels. HA-Ca_V_1.2_e22_ or Ca_V_1.2_e21+22_ were co-transfected with α_2_δ and β_2a_ subunit in HEK293 cells with or without MG132 treatment. β_2a_ subunit was cloned in pIRES2-EGFP (as an indicator of β_2a_ subunit expression). Immunostaining of total Ca_V_1.2 channels and confocal imaging were performed after 48 h transfection. (**A**) β_2a_ subunit-expressing cells showed up-regulation of total HA-Ca_V_1.2_e22_ channels with or without MG132 treatment, compared to cells not expressing β_2a_ subunits. (**B**) Total Ca_V_1.2_e21+22_ channels were not markedly altered in β_2a_ subunit-expressing cells with or without MG132 treatment. Scale bar, 20 μm.

To further confirm the roles of Ca_V_β subunits in regulation of Ca_V_1.2_e21+22_ channel function, confocal microscopy was performed to image and evaluate the total expression of Ca_V_1.2_e21+22_ channels with or without β_2a_ subunits. As previously reported^4^, compared to cells that did not express β_2a_ subunits, β_2a_ subunit-expressing cells showed increased expression level of total HA-Ca_V_1.2_e22_ channels with or without MG132 treatment (Fig. 4A), which is consistent with the finding that ubiquitination of HA-Ca_V_1.2_e22_ channels was significantly reduced in the presence of β_2a_ subunits under MG132 treatment (Fig. 3F,G). However, in line with the results as shown in Fig. 3B,C, the total expression of Ca_V_1.2_e21+22_ channels was not markedly increased in β_2a_ subunit-expressing cells with or without MG132 treatment (Fig. 4B), which also further supported that β_2a_ subunits did not significantly prevent the ubiquitination of Ca_V_1.2_e21+22_ channels (Fig. 3F,G).

### Ca_V_1.2_e21+22_ channels down-regulated expression of Ca_V_1.2_e22_ channels in a dominant-negative manner

**Figure 5:**
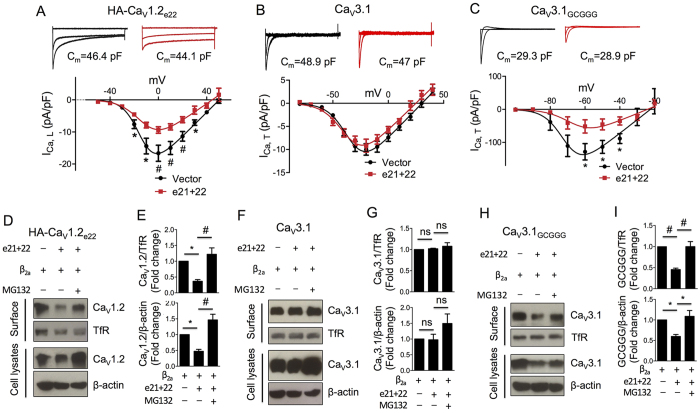
Ca_V_1.2_e21+22_ channels produce dominant-negative effects on L-type Ca_V_1.2 channels, but not on T-type Ca_V_3.1 channels. Ca_V_1.2_e21+22_ channels were co-transfected at a ratio of 1:1 with HA-Ca_V_1.2_e22_, Ca_V_3.1 or chimeric Ca_V_3.1_GCGGG_ channels containing Ca_V_1.2 I-II loop in HEK293 cells with or without MG132 treatment. As control, Ca_V_1.2_e21+22_ channels were replaced with pcDNA3 vectors for co-transfection. I-V curves were obtained in an external solution containing 1.8 mM Ca^2+^. For western blot assays, cells were biotinylated for surface proteins 36 hrs after transfection and then lysed for analysis. (**A**–**C**) Effects of Ca_V_1.2_e21+22_ channels on the current density of wild-type Ca_V_1.2_e22_ channels (Vector, n = 19; e21 + 22, n = 18), Ca_V_3.1 channels (Vector, n = 9; e21 + 22, n = 11) or the chimeric Ca_V_3.1_-GCGGG_ channels (Vector, n = 10; e21 + 22, n = 9). *p < 0.05, ^#^*p* < 0.01. (**D**–**I**) Effects of Ca_V_1.2_e21+22_ channels on the surface and total expression levels of HA-Ca_V_1.2_e22_ channels (**D**,**E**), Ca_V_3.1 channels (**F**,**G**) or chimeric Ca_V_3.1_GCGGG_ channels (**H**,**I**, n = 3). Transferrin receptor (TfR) was used as surface protein loading control. e22, wild-type HA-Ca_V_1.2_e22_ channel. e21 + 22, aberrant Ca_V_1.2_e21+22_ channel. Data were shown as mean ± SEM, ns, non-significant, *p < 0.05, ^#^*p* < 0.01, 1-way ANOVA with post hoc Bonferroni’s test was performed for multiple comparisons.

Based on the observed stronger interaction between Ca_V_1.2_e21+22_ channels and β_2a_ subunit, we hypothesized that Ca_V_1.2_e21+22_ channels may modulate the function of wild-type Ca_V_1.2 channels by depleting or competing for free β_2a_ subunits. To test this hypothesis, Ca_V_1.2_e21+22_ channels were co-transfected with Ca_V_β-dependent HA-Ca_V_1.2_e22_ channels or Ca_V_β-independent Ca_V_3.1 channels in HEK 293 cells. As measured in external solution containing 1.8 mM Ca^2+^, the current density of Ca_V_1.2 channels at 0 mV was lowered from −16.7 ± 2.5 pA/pF to −9.3 ± 1.0 pA/pF (*p* = 0.007, Fig. 5A). In contrast, the current density of Ca_V_3.1 channels at −20 mV remained unchanged (−9.9 ± 1.2 pA/pF vs. −9.2 ± 1.4 pA/pF, *p* = 0.671, Fig. 5B). To further investigate whether this dominant-negative effect on Ca_V_1.2 channels is due to reduction of free Ca_V_β subunits by Ca_V_1.2_e21+22_ channels, the Ca_V_1.2 I-II loop containing the AID domain that binds Ca_V_β subunit was substituted into Ca_V_β-independent Ca_V_3.1 channel to generate a chimeric Ca_V_3.1_GCGGG_ channel. Compared to Ca_V_3.1 channels, the chimeric channels displayed a dramatic increase in current amplitude and a 40 mV leftward shift in the I–V relationship (Fig. 5B,C) as previously reported^21^. Upon co-expression with Ca_V_1.2_e21+22_ channels, the current density of Ca_V_3.1_GCGGG_ channels at −60 mV was significantly reduced from −128.4 ± 22.2 pA/pF to −51.6 ± 16.8 pA/pF (Fig. 5C).

To further delineate the mechanisms underlying the differential regulation of current density by Ca_V_1.2_e21+22_ channels, the total and surface expression levels of all the three calcium channels were examined in HEK 293 cells co-transfected with β_2a_ subunit. As expected, Ca_V_1.2_e21+22_ channels did not affect the surface and total expression levels of Ca_V_3.1 channels (Fig. 5F,G). However, co-expression of Ca_V_1.2_e21+22_ channels significantly reduced the surface (upper panel, Fig. 5D,E) and total (lower panel, Fig. 5D,E) levels of Ca_V_1.2 channels and the chimeric Ca_V_3.1_GCGGG_ channels (Fig. 5H,I). The reduction in expression levels of Ca_V_β-dependent channels was prevented by MG132 treatment indicating proteasomal degradation of those channels co-expressed with the Ca_V_1.2_e21+22_ isoform (Fig. 5D,E).

### Ca_V_1.2_e21+22_ channels enhanced ubiquitination of Ca_V_β-binding calcium channels

**Figure 6:**
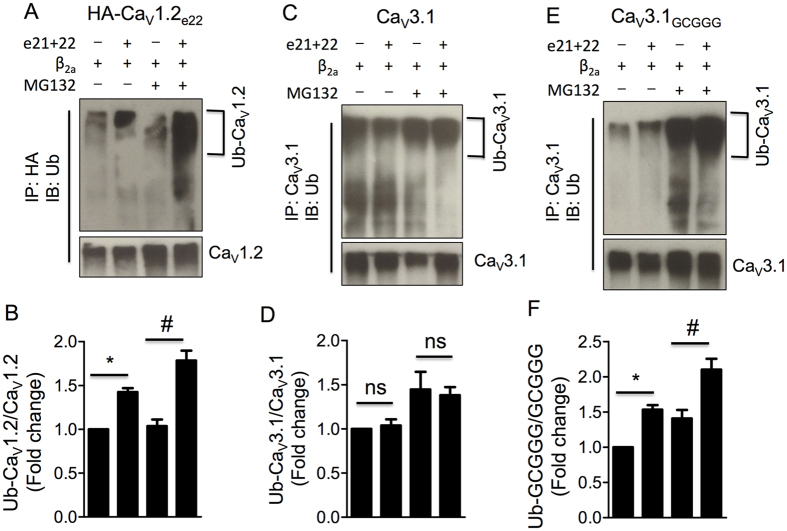
Ca_V_1.2_e21+22_ channels enhance ubiquitination of L-type Ca_V_1.2 channels, but not T-type Ca_V_3.1 channels. Ca_V_1.2_e21+22_ channels were co-transfected at a ratio of 1:1 with HA-Ca_V_1.2_e22_, Ca_V_3.1 or the chimeric Ca_V_3.1_GCGGG_ channels in HEK293 cells with or without MG132 treatment. Anti-HA and anti-Ca_V_3.1 were used to pull down the protein complexes from cell lysates for ubiquitnation analysis of HA-Ca_V_1.2_e22_ (**A**,**B**), wild-type Ca_V_3.1 channels (**C**,**D**) or chimeric Ca_V_3.1_GCGGG_ channels (**E**,**F**) with or without MG132 treatment (n = 3). e22, HA-Ca_V_1.2_e22_ channel. e21 + 22, Ca_V_1.2_e21+22_ channel. Ub-Ca_V_1.2, ubiquitinated Ca_V_1.2 channels. Data were shown as mean ± SEM, ns, non-significant, *p < 0.05, ^#^*p* < 0.01, 1-way ANOVA with post hoc Bonferroni’s test was performed for multiple comparisons.

As most proteasomal degradation involves ubiquitin, the ubiquitination levels of all the three calcium channels were evaluated in HEK 293 cells co-transfected with Ca_V_1.2_e21+22_ channels. As shown by western blot analyses, the relative intensity of ubiquitinated Ca_V_1.2 channels (Ub-Ca_V_1.2) to total Ca_V_1.2 channels was greatly enhanced by the presence of Ca_V_1.2_e21+22_ channels (*p* = 0.015, Fig. 6A,B), and the increase in ubiquitination of Ca_V_1.2 channels was augmented by MG132 treatment (*p* = 0.007, Fig. 6A,B). In contrast, the ubiquitination of Ca_V_3.1 channels was not affected by the presence of Ca_V_1.2_e21+22_ channels or MG132 treatment (Fig. 6C,D). While introduction of the Ca_V_β-binding domain into this Ca_V_β-independent channel markedly increased its ubiquitination by Ca_V_1.2_e21+22_ channels (Fig. 6E,F). These results suggested that the augmentation of ubiquitination of calcium channels by Ca_V_1.2_e21+22_ channels is attributed to the Ca_V_β-binding domain.

### Ca_V_1.2_e21+22_ channels competed for Ca_V_β subunits with Ca_V_1.2 channels

**Figure 7:**
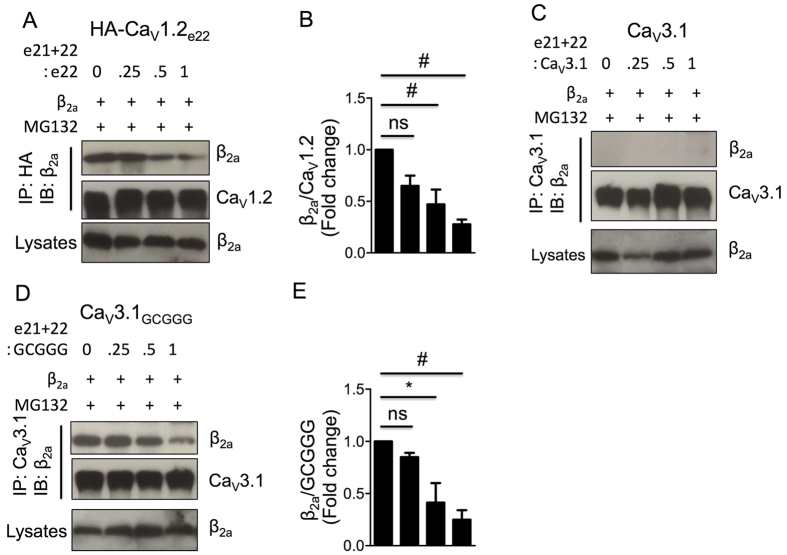
Ca_V_1.2_e21+22_ channels compete for β_2a_ subunit with L-type Ca_V_1.2 channels, but not with T-type Ca_V_3.1 channels, in a dose-dependent manner. Ca_V_1.2_e21+22_ channels were co-transfected at indicated ratios with HA-Ca_V_1.2_e22_, Ca_V_3.1 or chimeric Ca_V_3.1_GCGGG_ channels in HEK 293 cells treated with MG132. Anti-HA and anti-Ca_V_3.1 were used for co-immunoprecipitation. Effects of Ca_V_1.2_e21+22_ channels on the binding of β_2a_ subunits to HA-Ca_V_1.2_e22_ (**A**,**B**), Ca_V_3.1 channels (**C**) or chimeric Ca_V_3.1_GCGGG_ channels (**E**) were analyzed by Western blotting (n = 3). e22, wild-type HA-Ca_V_1.2_e22_ channel. e21 + 22, aberrant Ca_V_1.2_e21+22_ channel. Data were shown as mean ± SEM, ns, non-significant, *p < 0.05, ^#^*p* < 0.01, 1-way ANOVA with post hoc Bonferroni’s test was performed for multiple comparisons.

To substantiate the notion that the reduced expression level and increased ubiquitination of Ca_V_1.2 channels by Ca_V_1.2_e21+22_ channels were due to competition with Ca_V_1.2 channels for available Ca_V_β subunits, Ca_V_1.2_e21+22_ channels were co-transfected with Ca_V_1.2, Ca_V_3.1 or the chimeric Ca_V_3.1_GCGGG_ channels at a molar ratio of 0, 1/4, 1/2 or 1 in HEK 293 cells treated with MG132. As indicated by Western blot, the relative intensity of β_2a_ subunit to Ca_V_1.2 channels was gradually reduced with increase of Ca_V_1.2_e21+22_ channels (Fig. 7A,B). While no β_2a_ subunit was co-immunoprecipitated with wild-type Ca_V_3.1 channels (Fig. 7C) the relative intensity of β_2a_ subunit to chimeric Ca_V_3.1_GCGGG_ channels was clearly attenuated by Ca_V_1.2_e21+22_ channels in a dose-dependent manner (Fig. 7D,E).

## Discussion

This study identified a novel alternatively spliced isoform of Ca_V_1.2 channels, Ca_V_1.2_e21+22_. The expression of Ca_V_1.2_e21+22_ diminishes during postnatal cardiac maturation and re-emerges in pressure-overload induced cardiac hypertrophy. This fetal-like alternative splicing pattern of Ca_V_1.2 channels in the hypertrophied heart is in agreement with a recent report that alternative splicing events in response to TAC displayed reciprocal expression changes during postnatal cardiac development versus heart failure^22^. Despite its physiological significance during cardiac maturation, the role of the re-emergence of Ca_V_1.2_e21+22_ in response to cardiac stress is unknown.

Mutations in Ca_V_1.2 channels are associated with multiple heart diseases including Timothy syndrome that is characterized by a long QT interval and ventricular arrhythmia due to sustained activation of Ca_V_1.2 channels^23^,^24^ and Brugada syndrome that is notable for a short QT interval and sudden cardiac death due to inactivation of Ca_V_1.2 channels^25^. While the role of Ca_V_1.2 channels in electrical heart diseases is well known, its role in mechanical or structural heart diseases remains less understood. In failing human or animal hearts, the density and currents of Ca_V_1.2 channels were reportedly reduced^26^ or unchanged^27^. In causal studies with genetic modified animals, the conclusion is so far controversial. Increase in Ca^2+^ influx through Ca_V_1.2 channels by cardiac specific over-expression of β_2a_ subunit^7^ or α_1C_ subunit^28^ in mice was reported to induce cardiac hypertrophy and cardiomyopathy. Unexpectedly, decrease in Ca^2+^ influx through Ca_V_1.2 channels in α_1C_+/− mice resulted in a similar phenotype^8^. The disparities might be attributed to activation of calcineurin activation or neurohumoral effects^8^. Alternatively, it might be partly explained by the existence of two distinct subsets of the channels^24^,^29^,^30^: One subset assembled in the T-tubules for calcium-induced calcium release with ryanodine receptors for excitation-contraction coupling^31^,^32^, and the other subset (~50% in mice)^33^ enriched in caveolae to activate the transcription factor NFAT (nuclear factor of activated T cells) for cardiac hypertrophy. In line with this notion, we and Goonasekera *et al.* found that the protein level (Fig. 2) and activity of Ca_V_1.2 channels were reduced in pressure overload-induced hypertrophic hearts^23^. Detailed analysis showed that the density and current of Ca_V_1.2 channels both significantly declined in the isolated cardiomyocytes from those failing hearts. Although it is hard to prove which subset of channels decreased, according to their distinct functions, one may suspect that the caveolae-localized channels were affected the most in such a scenario.

One crucial question following Goonasekera’s study is how the channel expression and activity was reduced in response to pressure overload. As alternative splicing occurs frequently at 19 out of 55 exons that constitute the Ca_V_1.2 gene, *Cacna1c*, in rodent heart and artery^34^,^35^, and some alternatively spliced isoforms were suggested to dominant-negatively suppress expression and channel conductivity of calcium channels^14^,^36^, we therefore hypothesized that the fetal splice variant Ca_V_1.2_e21+22_ may reemerge in adult heart in response to cardiac stress, based on the recent findings by Gao and colleagues^22^, and disrupt the expression and activity of Ca_V_1.2 channels. In agreement with Goonasekera’s findings, the total expression of Ca_V_1.2 channels and Ca_V_β_2_ subunits were significantly reduced in left ventricles in response to TAC surgery (Fig. 2F–H). More importantly, the abundance of the Ca_V_1.2_e21+22_ splice variant in mouse left ventricles was gradually increased up to 12.5 folds within 14 days after TAC (Fig. 2), and also elevated in left ventricles of DCM patients (Supplementary Fig. S2). Aberrant splicing of Ca_V_1.2 channels was reported, though in very few studies, in cardiovascular diseases. For example, the abundance of exon 31- and exon 32-containing Ca_V_1.2 isoforms significantly changed in end stage failing human hearts^17^ and smooth muscle Ca_V_1.2 channel including exon 21 was completely replaced by a single isoform containing alternative exon 22 in human atherosclerosis^18^. However, the patho-physiological significance of these splicing events is unknown.

In the present study, we demonstrated that Ca_V_1.2_-e21+22_ splice variant was retained intracellularly and it did not conduct Ca^2+^ (Fig. 3B,C). Strikingly, stronger binding to Ca_V_β subunit did not increase the accumulation and trafficking of Ca_V_1.2_e21+22_ channels to cell surface, which appears contradictory to the conceptual model proposed by the Colecraft’s group^21^. In that model, it was proposed that following Ca_V_β interaction, a conformational rearrangement of the C-terminus attenuates the strength of ER retention signals within the C-terminus relative to the export signals found within the I-II loop. The net result would be enhanced trafficking to plasma membrane^21^. However, the C-terminus of rabbit Ca_V_2.1 channels was shown to specifically interact with Ca_V_β_4_ subunit; meanwhile it also displayed a lower binding to Ca_V_β_2_ subunit^37^. In addition, Qin *et al.* also proposed that Ca_V_β_2a_ inhibition of the inhibitory effect of Gβγ (G protein βγ dimers) on R-type Ca_V_2.3 channel activity could be explained by the competitive displacement of Gβγ from its C-terminal binding site by the Ca_V_β_2a_ subunit^38^. Based on these findings, we speculate that the presence of two transmembrane segments, exons 21 and 22, in Ca_V_1.2_e21+22_ channel may induce conformational changes in the C-terminus, which may lead to a stronger binding to Ca_V_β subunit and prevent the Ca_V_β-dependent conformational rearrangement of the C-terminus as proposed in wild-type Ca_V_1.2 channel. As a result, Ca_V_1.2_e21+22_ channels fail to be transported to cell membrane and are trapped in ER to be degraded as misfolded proteins.

Ca_V_1.2_e21+22_ channels suppressed expression of Ca_V_1.2 channels via a dominant-negative mechanism. It has been shown that the misfolded calcium channels could drive wild-type channels toward proteasomal degradation, leading to a significant dominant-negative effect^14^,^36^. For example, the truncated variants of P/Q-type Ca_V_2.1 channels could drive wild-type Ca_V_2.1 channels into ER-associated degradation system by directly binding to domain I-II region^36^, which required an intact N-terminus^39^. However, our data did not support a direct interaction between Ca_V_1.2_e21+22_ channels and wild-type Ca_V_1.2 channels. Furthermore, the obvious dominant-negative effect of Ca_V_1.2_-e21+22_ channels on the chimeric Ca_V_3.1_GCGGG_ channels (Fig. 5) excluded a major role of the N-terminus suggested for Ca_V_2.1 channels^39^. Therefore the dominant-negative effect of Ca_V_1.2_e21+22_ channels is likely attributed to a disparate mechanism. As Ca_V_1.2_e21+22_ channels showed significantly stronger binding to Ca_V_β subunits than that of the wild type channels, this aberrant isoform may act as a Ca_V_β subunit trap by competing for free Ca_V_β subunits. Dose-dependent inhibition of the interaction between Ca_V_β subunits and Ca_V_1.2 or Ca_V_3.1_GCGGG_ channels by Ca_V_1.2_e21+22_ channels further supported this hypothesis. The competition for Ca_V_β subunits by Ca_V_1.2_e21+22_ channels may result in a shortage of free Ca_V_β subunits for Ca_V_1.2 channels and eventually lead to impaired membrane targeting, elevated ubiquitination and thereby increased degradation of Ca_V_1.2 channels. Accordingly, the enhanced ubiquitination and diminished expression^8^ of Ca_V_1.2 channels in the hypertrophied mouse heart induced by pressure overload are presumably caused by the reemergence of Ca_V_1.2_e21+22_ channels under stress (Fig. 2E). However, it is noteworthy that there are two major isoforms for Ca_V_β subunits (β_2_ and β_3_ subunit), quantitatively in the order of β_2b_ > β_3_ > β_2a_ in the heart^40^. Thus whether the competition for Ca_V_β subunits by Ca_V_1.2_e21+22_ channels is Ca_V_β isoform-dependent in cardiomyocytes will warrant further study.

Altogether, we may not anticipate that overexpression of Ca_V_1.2_e21+22_ channels in the heart will induce cardiac hypertrophy, rather Ca_V_1.2_e21+22_ channels could dominant-negatively disturb particularly the caveolae-localized Ca_V_1.2 channels and activate the calcineurin/NFAT in response to hypertrophic stresses. Nevertheless, it is tempting to speculate that, in the patients suffering aortic stenosis or severe hypertension, Ca_V_1.2_e21+22_ channels may reemerge in the heart and disturb the expression and activity of Ca_V_1.2 channels, in particular of those channels localized in the caveolae, and consequently lead to cardiac hypertrophy.

In conclusion, we have identified and functionally characterized a naturally occurring fetal splice variant of Ca_V_1.2 channels (Ca_V_1.2_e21+22_ channels) that reemerged in adult mouse heart under stress and consequently disturbed the expression and activity of Ca_V_1.2 channels. In addition, we demonstrated that this splice isoform augmented the ubiquitination of Ca_V_1.2 channels for proteasomal degradation, impaired membrane targeting of the channels and reduced the channel expression and activity by competing for available Ca_V_β subunits. These data may provide a new insight of the dynamics of Ca_V_1.2 channels at molecular level in the setting of cardiac hypertrophy.

## Methods

### Study approval

All human heart samples obtained from Cardiovascular Research Institute, National University of Singapore (NUS), were de-identified and pre-existing. All the experiments on the human heart tissues were performed in accordance with guidelines and protocols approved by the NUS Institutional Review Board (Reference code: 12-405). One normal human heart total RNA was purchased from Clontech (636532). All animal experiments were performed in accordance with guidelines and protocols approved by the Institutional Animal Care and Use Committee of National University of Singapore.

### Induction of cardiac hypertrophy in mice

C57BL/6 mice were purchased from Jackson Laboratory and maintained at the Comparative Medicine Animal Vivarium at National University of Singapore. Experiments were carried out on adult male C57BL mice (10–12 weeks). Mice were anesthetized with a cocktail of 0.5 mg/kg Domitor, 5 mg/kg Dormicum and 0.05 mg/kg Fentanyl via intra-peritoneal injection, intubated and ventilated with a rodent ventilator (Harvard Apparatus). Transverse aortic constriction (TAC) was performed as previously described^41^. Briefly, the transverse aortic arch was exposed by a median sternotomy and bonded against a blunt 27-gauge needle with a 7-0 suture followed by prompt removal of the needle. Sham operated mice underwent the same procedure without aortic binding. Left ventricles were isolated for qPCR analysis of Myh6 and Myh7, transcript screening of exon 21 + 22 or biochemical analysis of of Ca_V_1.2 channels and Ca_V_β_2_ subunits. Echocardiography was performed with Vevo 2100 from Visualsonics.

### Transcript screening

As previously described^14^, total RNA was isolated using Trizol method total RNA was isolated using Trizol method from neonatal rat hearts, or left ventricles of adult rats, mice or patients with DCM or ICM. Then first strand cDNA was synthesized with Superscript II and oligo(dT)18 primers. PCR products (Primers for screening in rat heart: sense primer 5′-ACACTGCAGGTGAAGAGGATG-3′ and antisense primer 5′-TTTCCCTTGAAGAGCTGGACC-3′. For mouse heart: sense primer 5′-GAGCTGCACCTTAAGGAAAAGG-3′ and antisense primer 5′-GGATGCCAAAGGAGATGAGG-3′. For human heart: sense primer 5′-CCACCGCATTGTCAATGACAC-3′ and antisense primer 5′-CACGATGTTCCCGATGGTC-3′) were cloned into the pGEM-T Easy vector. Following transformation, each transformant was picked and grown in a single well in a 96-well plate. Colony PCR was performed with the same set of primers and conditions to identify the component of exons in each colony. 192 colonies were selected for each sample and at least 5 clones from each cDNAs group were sequenced to verify the exon specific PCR results.

### DNA constructs

β_2a_ and α_2_δ subunits have been described previously^42^. Chimeric Ca_V_3.1_GCGGG_ channel^21^ was kindly provided by A/Prof Henry Colecraft from Columbia University, and rat HA-Ca_V_1.2_e22_ channel (wild type Ca_V_1.2 channel)^43^ was from Prof Emmanuel Bourinet from Institut de Génomique Fonctionnelle. The cloning of rat Ca_V_1.2_e21+22_ channel was achieved by inserting a PCR fragment containing exon 21 + 22 into the wild-type channel using NotI and KpnI sites.

### Cell culture and transfection

HEK293 cells were cultured in Dulbecco’s Modified Eagle Medium (DMEM, Gibco) containing 10% fetal bovine serum (Gibco) and 1% penicillin–streptomycin and maintained at 37 °C in a humidified atmosphere containing 95% air and 5% CO_2_. For co-immunoprecipitation experiments, if not specified, HEK293 cells were cultured in 6-well plates, and were transiently transfected with different calcium channels, β_2a_ subunit, α_2_δ subunit using calcium phosphate methods. In some experiments, cells were treated with a proteasomal inhibitor MG132 (2.5 μM) for 16 hrs at 24 hrs after transfection in order to prevent the proteasomal degradation of calcium channels. For whole cell patch-clamp recordings, HEK293 cells cultured on the coverslips coated with poly-D-lysine in 35 mm dishes were transiently transfected with different calcium channels at a molar ratio of 1:1 unless otherwise stated.

### Co-immunoprecipitation

Co-immunoprecipitation was performed as described previously with modification. In brief, proteins harvested from transfected HEK293 cells were incubated with primary antibodies overnight at 4 °C with gentle rotation, followed by incubation with 20 μl of protein A/G agarose (Pierce) for another 1 h at 4 °C. The beads were washed 3 times using cold PBS and then denatured in 2X SDS sample loading buffer by boiling at 95 °C for 10 min. Proteins were then used for western blot analysis.

### Surface protein biotinylation

To determine the level of Ca_V_1.2_e21+22_ channels localized on the cell surface, Ca_V_1.2_e21+22_ channels were biotinylated using an EZ-Link^TM^ Sulfo-NHS-Biotinylation Kit (Pierce) as previously described with modifications^44^. Briefly, cells were incubated with 0.25 mg/ml Biotin for 1h at 4 °C. Unbound biotin was removed by incubation with quenching buffer for 20 min and washed by PBS buffer. After measurement of protein concentration with Bradford assay, cell lysates were incubated with NeutrAvidin (Pierce) overnight to pull down the biotinylated surface proteins. The precipitates were boiled in 2X sample loading buffer to elute Avidin-bound for SDS-PAGE analysis. GAPDH was used as a cytoplasmic marker to assess whether the surface biotinylated fractions include cytoplasmic channels (Data were not included).

### Ubiquitination assay

Ca_V_1.2_e21+22_ channels were transiently transfected with HA-Ca_V_1.2_e22_, wild-type Ca_V_3.1 or chimeric Ca_V_3.1_GCGGG_ channels in HEK 293 cells. Twenty-four hours after transfection, cells were treated with MG132 (2.5 μM) overnight and then lysed in PBS buffer containing 1% SDS and 1 mM EDTA. Cell lysates were boiled for 5 min at 95 °C, votexed for 10 sec, and then boiled for another 3 min at 95 °C. Ubiquitinated substrates in the supernatant were immunoprecipitated with anti-Ca_V_3.1 or anti-HA, washed 3 times with cold PBS buffer, and resolved by 8% SDS-PAGE gel.

### Western blot

Cells were harvested using lysis buffer (50 mM Tris, 150 mM NaCl, 1 mM EDTA, 1% Triton X-100, pH 7.4) containing protease inhibitor cocktails (Roche) at 48 hrs after transfection. After measurement of protein concentration, cell lysates were separated by 8% SDS-PAGE for 50 min at 150 V and transferred onto PVDF membrane at 30 V overnight at 4 °C. Subsequently, the membrane was blocked with 5% non-fat milk for 1h at room temperature and then incubated overnight at 4 °C with primary antibodies: rabbit anti-Ca_V_1.2 (1:1000, ACC-003, Alomone), anti-Ca_V_3.1 (1:1000, ACC-021), rabbit anti-β_2_ (1:1000, ACC-105), mouse anti-ubiquitin (1:1000, 13–1600, Invitrogen), rabbit anti-HA (1:1000, 71–5500), mouse anti-TfR (1:1000, 13–6800), mouse anti-β-actin (1:5000, A1978, Sigma) or mouse anti-GAPDH (1:2000, G8795). The membrane was washed three times with TBS-T buffer and then incubated with corresponding HRP-conjugated secondary antibodies (1:5000) for 1h at room temperature. After washing, proteins were detected using West Pico or Femto Chemiluminescent Substrate (Pierce). The blots were analyzed with ImageJ software (NIH).

### Confocal imaging

HA-Ca_V_1.2_e22_ or Ca_V_1.2_e21+22_, α_2_δ and β_2a_ subunit in pIRES2-EGFP as an indicator of β_2a_ subunit expression were co-transfected in HEK293 cells cultured in 35 mm dish using calcium phosphate method. As described previously^4^, 48 h after transfection, cells were passaged to 2 wells with coated coverslips in 12-well plate, followed by 10 μM MG132 treatment for 2 h with cells in one of the wells. After that, cells were washed by cold PBS containing 5% FBS and fixed in 4% paraformaldehyde for 15 min. Following permeablization using 0.2% Tween-20/PBS for 15 min and blocking by 10% FBS/PBS for 20 min, cells were stained with rabbit anti-Ca_V_1.2 (1:100, Alomone) at 37 °C for 60 min. Alexa Fluor 594-conjugated goat α-rabbit IgG antibody (Molecular Probes, 1:300) was used as secondary antibody to incubate with cells for 60 min in room temperature.

Cells were imaged using a Zeiss LSM-510 Meta confocal microscope with a 63 × 1.4NA oil immersion lens in the inverted position. EGFP was visualized by excitation with an argon laser (488 nm) and emission detected using a long-pass 530-nm filter. AF-594 antibody was visualized by excitation with a HeNe laser (543 nm) and emission detected using a 585–615 nm bandpass filter. Image acquisition was performed with identical gain, contrast, laser excitation, pinhole aperture and laser scanning speed for 3 rounds of cultures.

### Electrophysiological recordings

As previously described, patch-clamp recordings were performed at 24–72 hrs after transfection using an Axopatch 200B amplifier (Molecular Device). The external solution contained 144 mM TEA-MeSO_3_, 10 mM HEPES, 1.8 mM CaCl_2_ (pH 7.4 adjusted with CsOH and osmolarity 300–310 mOsm with glucose). The internal solution contained 138 mM Cs-MeSO_3_, 5 mM CsCl, 0.5 mM EGTA, 10 mM HEPES, 1 mM MgCl_2_, 2 mg/ml Mg-ATP (pH 7.3 adjusted with CsOH and osmolarity 300–310 mOsm with glucose). To determine the whole cell current-voltage (*I-V*) relationships, currents were recorded by holding the cell at −90 mV (or −100 mV for Ca_V_3.1_GCGGG_ channel) before stepping to various potentials from −90 to 60 mV (or −100 to 40 mV for Ca_V_3.1_GCGGG_ channel) over 900 ms. The *I-V* curve was fitted with the equation: *I*_Ca_ = *G*_max_(*V* − *E*_rev_)/(1 + exp((*V* − *V*_1/2_)/*k*), where *G*_max_ is the maximum conductance; *E*_rev_ is the reversal potential; *V*_1/2_ is the half-activation potential; and *k* is the slope.

### Statistics

All results were presented as mean ± SEM. Student’s *t* test was performed to compare two independent groups and one-way ANOVA followed by Bonferroni post hoc test was used for multiple group comparisons. *P* < 0.05 was considered significant.

## Additional Information

**How to cite this article**: Hu, Z. *et al.* Aberrant Splicing Promotes Proteasomal Degradation of L-type Ca_V_1.2 Calcium Channels by Competitive Binding for Ca_V_β Subunits in Cardiac Hypertrophy. *Sci. Rep.* **6**, 35247; doi: 10.1038/srep35247 (2016).

## Supplementary Material

Supplementary Information

## Figures and Tables

**Table 1:** Echocardiographic characteristics of mice 0 or 14 days after TAC surgery.

	Baseline	Day 14 post-TAC
Heart rate (bpm)	421 ± 22	479 ± 21*
LVAW; d (mm)	0.74 ± 0.03	1.15 ± 0.04^§^
LVAW; s (mm)	1.00 ± 0.05	1.41 ± 0.06^§^
LVPW; d (mm)	0.55 ± 0.02	0.96 ± 0.06^§^
LVPW; s (mm)	0.70 ± 0.04	1.10 ± 0.07^§^
LVID; d (mm)	4.25 ± 0.08	4.11 ± 0.12
LVID; s (mm)	3.35 ± 0.15	3.49 ± 0.13
LVEF (%)	49.53 ± 2.90	33.86 ± 2.53^#^
FS (%)	25.05 ± 1.82	16.18 ± 1.45^#^
CO (ml/min)	16.68 ± 1.11	12.08 ± 1.04^#^
LV mass (mg)	74.29 ± 2.52	135.50 ± 11.06^§^

Echocardiography of mice 0 or 14 days after TAC surgery (n = 6). LVAW, left ventricle anterior wall thickness at end diastole (d) or end systole (s); LVPW, left ventricle posterior wall thickness; LVID, left ventricle internal dimension; LVEF, left ventricle ejection fraction; FS, fraction shortening; CO, cardiac output. *p < 0.05, ^#^*p* < 0.01, ^§^*p* < 0.001.
