# Supplementary material for: Aberrant Splicing Promotes Proteasomal Degradation of L-type CaV1.2 Calcium Channels by Competitive Binding for CaVβ Subunits in Cardiac Hypertrophy
Source: Sci Rep. 2016 Oct 12;6:35247. doi: 10.1038/srep35247 (PMC5059693; doi:10.1038/srep35247)
Supplement: Supplementary Information [file srep35247-s1.pdf]

Supplementary Information

Aberrant Splicing Promotes Proteasomal Degradation of L-type $Ca_V1.2$ Calcium Channels by Competitive Binding for $Ca_V\beta$ Subunits in Cardiac Hypertrophy

Zhenyu Hu, Jiong-Wei Wang, Dejie Yu, Jia Lin Soon, Dominique PV de Kleijn, Roger Foo, Ping Liao, Henry M. Colecraft, Tuck Wah Soong

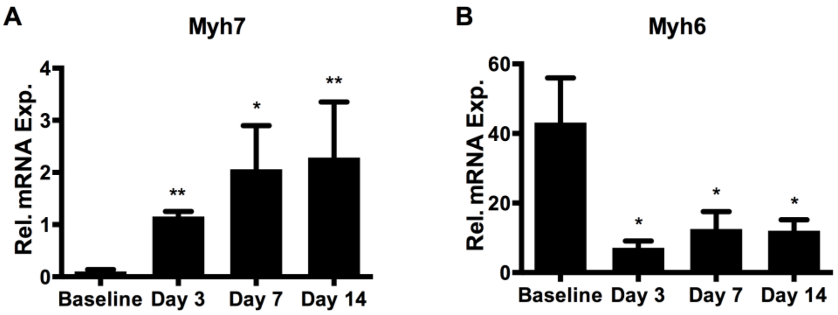

Figure S1. Expression of hypertrophic genes in the mouse hearts in response to TAC. Relative expression of mRNA to 18s RNA (internal control) was quantified by qPCR with mouse left ventricle lysates at indicated time after TAC surgery. The increase in Myh7 and decrease in Myh6 indicate cardiac hypertrophy. The number of animals examined: Baseline n=6, Day 3 n=4, Day 7 n=5 and Day 14 n=6. Data were shown as mean ± SEM. Compared with baseline group, *p<0.05, **p<0.01.

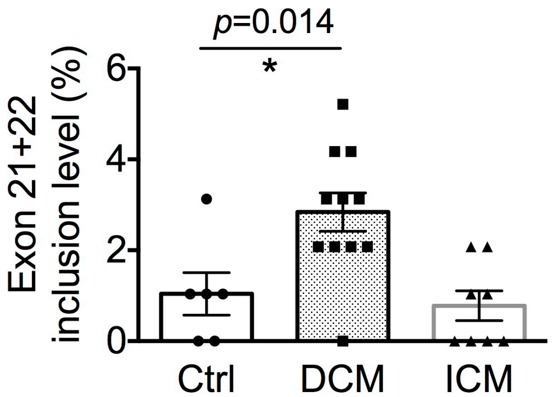

Figure S2. **Exons 21+22 inclusion level in left ventricles of human patients with or without dilated or ischemic cardiomyopathy**. Inclusion level of exons 21+22 was significantly higher in heart tissues from patients with dilated cardiomyopathy (DCM, 2.84%) than that in control heart tissue (1.04%). No elevation of inclusion level of exons 21+22 was observed in heart samples from patients with ischemic cardiomyopathy (ICM, 0.78%). The number of human samples examined: Ctrl, n=6; DCM, n=11; ICM, n=8. Data were shown as mean ± SEM. Compared with Ctrl group, *p<0.05.

Table S1. Patient characteristics for human heart samples

Group	Sample code	Age	Sex	Date Taken	Diagnosis summary	Pre-op medications
Control	2634	53	M		SAH	
	2824	48	M		ICB	
	2871	NA	NA			
	3074	47	M		Intraventricular haemorrhage	
	7454	NA	NA			
	Clontech	Cat# 636532		Normal human heart total RNA		
DCM	TB12.0122	61	M	26/01/2012	Non-ischemic cardiomyopathy - diagnosed 1997 (with LV Thrombus) Non sustained VT, ICD implant 1999, Upgrade to CRTD 2002, Box change Sept 2010 (occluded left subclavian venous system), Chronic renal impairment	Digoxin, Bumetanide, Hydralazine, EPO, Candesartan, Bisoprolol, Sanatogen Gold, Warfarin, Simvastatin
	TB12.0240	68	M	20/02/12	Dilated cardiomyopathy. Normal heart (echo) 2008. Progressive breathlessness since about Nov 11. Decompensated CCF / admitted Nov 11. LVDD 64mm / EF <25% (echo) Nov 2011 and Jan12. Trivial coronary disease (angiogram) Nov 2011. Inotrope dependant.	Dopamine, Adrenaline, Furosemide, Omeprazole, Amiloride, Spironolactone, Heparin
	TB11.0642	61	M	07/06/11	Dilated Cardiomyopathy Atrial standstill Permanent pacing	Bumetanide Amiloride Candesartan Carvedilol Thyroxine Allopurinol Amiodarone
	TB12.0754	43	M	31/05/12	Dilated Cardiomyopathy	Furosemide Digoxin Candesartan

					Normal Coronary artreries	Carvedilol Perindopril Spironolactone Warfarin
	TB11.0948	51	M	08/08/11	Dilated Cardiomyopathy	Other (see comments) Patient was transferred from another hospital and notes were not available to collect information.
	TB11.1027	64	F	25/08/11	Dilated Cardiomyopathy	NA
	TB12.1094	29	M	30/07/12	Dilated Cardiomyopathy	Furosemide Digoxin Candesartan Carvedilol Perindopril Spironolactone Warfarin
	TB12.1222	54	M	19/08/12	DCM; ICD Implant	Bisoprolol; Spironolactone; Ramipril; Bumetanide; Bendroflumethiazide; Warfarin
	TB11.1308	60	M	14/10/11	Dilated Cardiomyopathy	Docusate Metoclopramide Omeprazole Ondansetron Paracetamol Amiloride Laxido Amiodarone Dopamine Enoximone
	TB11.1626	45	M	06/12/11	Dilated Cardiomyopathy	Ramipril, Amiloride, Carvedilol, Bumetanide, Warfarin, Metolazone
	TB11.1730	41	M	21/12/11	Heart Failure Poor ventricular function, Hypotension, Preserved Renal Function	Perindopril, Bisoprolol, Bumetanide, Spironolactone
ICM	TB12.0533	52	M	17/04/12	ISCHEMIC HEART DISEASE; Prior STEMI Nov 2009, Rescue PCI, Severe LV systolic dysfunction, VT with Syncope, CRTD- 2009, Type II Diabetes	Atorvastatin, Valsartan, Eplerenone, Aspirin, Bisoprolol, Levothyroxine, Ranitidine, Heparin subcut 5000 units twice aday, Furosemide, Dopamine
	TB12.0858	50	M	20/06/12	NA	NA
	TB12.1144	45	M	06/08/12	MI may 2010;	Bumetanide;

					Heartware LVAD 26/08/10; ICD Medronic	Amiloride; Omeprazole; Bisoprolol; Aspirin; Candesartan; Simvastatin; Warfarin
	TB12.1253	52	M	23/08/12	ORIF # wrist 1983; MI 2002; CABG x3 2003; Bivent PPM 02/12/10; Asthma/COPD; IDDM (HbA1c 8.6%); Artheroscopy 1985; Angina	Ivabradine; Fluticasone; Furosemide; Aspirin; Atorvastatin; Nicorandil; Omeprazole; Novomix; Sertraline; NovoRapid; Valsartan; Carvedilol; Eplerenone; Tadalafil; Montelukast
	TB11.1260	51	M	06/10/11	Ischemic Heart Disease	Ramipril Furosemide Omeprazole Aspirin Warfarin Bisoprolol Ferrous Sulphate Paracetamol
	TB11.1265	51	F	10/10/11	Ischemic Heart Disease	Aspirin Ramipril Bumetanide Epleronone Carvedilol Lansoprazole Candesartan Digoxin Warfarin
	TB12.1375	60	M	14/09/12	ICD in situ, Drug eluting stents, thrombus not on warfarin as increased bleeding risk	Amiodarone, Epleronone, Amiloride, Lansoprazole, Perindopril, Atorvastatin, Ferrous Sulphate, Flavonate HCL, Furosemide
	TB11.1750	53	M	28/12/11	NYHA III, Pulmonary Hypertension, Non sustained VT on Holter, series of MI, CABG 2005,	Bumetanide, Epleronone, Ramipril, Bisoprolol, Lansoprazole, Prosigrel, Aspirin, Simvastatin, Amiodarone, Nitrazepam
